# TiCl_4_ Dissolved in Ionic Liquid Mixtures from Аb Initio Molecular Dynamics Simulations

**DOI:** 10.3390/molecules26010079

**Published:** 2020-12-26

**Authors:** Lars Esser, Roberto Macchieraldo, Roman Elfgen, Melanie Sieland, Bernd Michael Smarsly, Barbara Kirchner

**Affiliations:** 1 Mulliken Center for Theoretical Chemistry, University of Bonn, Beringstrasse 4+6, D-53115 Bonn, Germany; esser@thch.uni-bonn.de (L.E.); macchieraldo@thch.uni-bonn.de (R.M.); elfgen@thch.uni-bonn.de (R.E.); 2 Institute of Physical Chemistry, Justus Liebig University, Heinrich-Buff-Ring 17, D-35392 Giessen, Germany; Melanie.Sieland@phys.chemie.uni-giessen.de (M.S.); Bernd.Smarsly@phys.Chemie.uni-giessen.de (B.M.S.)

**Keywords:** ionic liquids, ab initio molecular dynamics simulations, TiO_2_, material synthesis in ionic liquids

## Abstract

To gain a deeper understanding of the TiCl${}_{4}$ solvation effects in multi-component ionic liquids, we performed ab initio molecular dynamics simulations of 1-butyl-3-methylimidazolium [C${}_{4}$C${}_{1}$Im]${}^{+}$, tetrafluoroborate [BF${}_{4}$]${}^{-}$, chloride [Cl]${}^{-}$ both with and without water and titanium tetrachloride TiCl${}_{4}$. Complex interactions between cations and anions are observed in all investigated systems. By further addition of water and TiCl${}_{4}$ this complex interaction network is extended. Observations of the radial distribution functions and number integrals show that water and TiCl${}_{4}$ not only compete with each other to interact mainly with [Cl]${}^{-}$, which strongly influences the cation-[BF${}_{4}$]${}^{-}$ interaction, but also interact with each other, which leads to the fact that in certain systems the cation-anion interaction is enhanced. Further investigations of the Voronoi polyhedra analysis have demonstrated that water has a greater impact on the nanosegregated system than TiCl${}_{4}$ which is also due to the fact of the shear amount of water relative to all other components and its higher mobility compared to TiCl${}_{4}$. Overall, the polar network of the IL mixture collapses by including water and TiCl${}_{4}$. In the case of [Cl]${}^{-}$ chloride enters the water continuum, while [BF${}_{4}$]${}^{-}$ remains largely unaffected, which deeply affects the interaction of the ionic liquid (IL) network.

## 1. Introduction

Over the years, ionic liquids (ILs) have been of scientific interest as solvents and reaction media, because they are governed by a wide variety of intermolecular interactions, ranging from weak (van der Waals) to strong (Coulomb) interactions. This leads to unique structures at the molecular level, e.g., the segregation into polar and non-polar domains called microheterogeneity [1,2,3,4,5,6]. Due to various physico-chemical properties for some ILs such as low vapor pressure [7,8], electrochemical, chemical and thermal stability and microheterogeneity, ionic liquids are possible candidates for many applications such as in electrochemistry [9,10,11,12,13,14,15,16,17,18,19] in extraction processes [20,21,22], and in material synthesis [23,24]. In particular, the nanosegregated structure of ILs underlines their ability as a reaction medium to act as a structure directing agent [25]. In general, ILs support the formation of liquid clathrates via IL mixtures with aromatic hydrocarbons [26,27,28]. They are suitable as organocatalysts, as they function as a source for N-heterocyclic carbene catalysis [29,30]. For example the catalysis of the ring opening for the polymerization of lactides [29,30] and much more are carried out with the aid of ILs. Furthermore, they can also be used for the synthesis of inorganic substances under moderate reaction conditions. These products can be kinetically stabilized and may not be synthesized in any other way [31,32,33,34,35,36,37,38,39,40,41].

Such structure directing effects are also observed in inorganic material synthesis [25,38,42]. Proof of these effects was provided by the synthesis of 3-D iodometallate networks like [Bu${}_{3}$MeN]${}_{3}$[cis-Bi${}_{3}$I${}_{12}$] and GeI${}_{4}$-I${}^{-}$ via asymmetric cations which are sterically hindering [43,44]. Well-known examples for the use of ILs in inorganic material synthesis are the ionothermal routes of zeolithe analogous framework compounds and metal-organic frameworks (MOFs). For this purpose large organic cations are used to induce structure directing effects [25,42]. Further examples are studied by Dehnen using compositions of [K${}_{4}$(H${}_{2}$O)${}_{3}$][Ge${}_{4}$Se${}_{10}$] in addition with SnCl${}_{4}$ under ionothermal conditions to synthesize extended chalcogenide structures of 3D- [Sn${}_{9}$Se${}_{20}$]${}^{4-}$ substructures. These structures might contain interesting physical properties such as semi-/photoconductivity, molecular trapping potential, ion transport capability or chemical and catalytic activities [41]. Another example is the synthesis of highly stoichiometric tellurium bromide (Te${}_{2}$Br) [45,46]. Previous synthesis routes suggested increased reaction temperatures above 400 K and lasted several weeks, while newly found routes using imidazolium-based ILs with aluminum tetrachloride (AlCl${}_{3}$) lasted only a few days at room temperature [45,46]. Tellurium has a large variety of polycations that can be obtained by IL-assisted synthesis [36,40,46,47,48,49,50,51].

Experimental work also focused on a special approach to synthesize bronze-type TiO${}_{2}$ in ILs under moderate conditions. They demonstrated that depending on the ratio of the two ILs [C${}_{16}$C${}_{1}$Im][Cl] and [C${}_{4}$C${}_{1}$Im][BF${}_{4}$], water addition and using titanium tetrachloride [TiCl${}_{4}$] as a precursor, besides the desired bronze phase (B), different TiO${}_{2}$-phases (anatase, rutile and a fluorine-rich phase) and crystallite sizes and morphologies occur [24]. A crucial question in all these applications is: “How are these liquids structured on the molecular level and what kind of properties follow them?” Tackling this question, the theoretical investigation is a necessity. In this regards joint approaches between experiment and theory were carried out for example by D’Angelo and Serva to investigate the structural properties of geminal di- and monocationic imidazolium based ILs with halides especially in mixtures with water [52,53].

Further insights into the chemical evolution of a system can be achieved by performing AIMD simulations, which give additional access to the electronic structure of the observed particles. For instance, the AIMD simulation of the material synthesis of Te${}_{4}$Br${}_{2}$ in a mixture of [C${}_{2}$C${}_{1}$Im][Cl]·1.3 AlCl${}_{3}$ indicated that the structural diffusion of chloroaluminate (III) units in ILs play a significant role in the synthesis of tellurium polycations, which has similarities to the Grotthuss mechanism in water [23]. Other theoretical studies based on AIMD simulations have been done previously in this respect to investigate Grotthuss-like mechanisms in ILs such as 1-ethyl-3-methylimidazolium-chloroaluminate-based protic ionic liquids [C${}_{2}$C${}_{1}$Im][AlCl${}_{4}$]·32AlCl${}_{3}$ in which large anions reassemble by structural diffusion over time [19,54,55]. Comparable mechanisms for other species have also been studied theoretically [56,57], like the studies of Hollóczki et al. of different types of inorganic chlorides in halide ILs, where in case of PCl${}_{3}$ highly effective anionic Grotthuss diffusion is observed and other examples such as iodide anion conduction in ILs with iodide/triiodide redox couples [58,59,60] and the CO${}_{2}$ transport in molten calcium carbonate [61,62].

In this work we analyze the effects of different solvent mixtures in order to understand the solvation effects therein. This comprises the IL mixture 1-butyl-3-methylimidazolium [C${}_{4}$C${}_{1}$Im]${}^{-}$, tetrafluoroborate [BF${}_{4}$]${}^{-}$, chloride [Cl]${}^{-}$ both with and without water and titanium tetrachloride TiCl${}_{4}$. For this purpose we perform molecular dynamics simulations to provide molecular level insight into the IL ion-interplay, the influence on the complex interaction network upon addition of TiCl${}_{4}$ and water.

The article is structured as follows: Section 2.1, Section 2.2, Section 2.3 and Section 2.4 dealt with the structural properties of the simulated systems, while Section 2.5 and Section 2.6 dealt with the dynamical properties. Section 2.1, Section 2.2 and Section 2.3 radial distribution functions (RDFs) and number integrals (NI) are created and the corresponding tables illustrate the most important points of the figures. Section 2.1, Section 2.2 and Section 2.3 are divided in such a way that each sub-point refers mainly to different interactions. Section 2.1 deals with the interactions between cations and anions and the corresponding hydrogen bonds. Section 2.2 deals with the effects on the solvation structures of the water in the investigated systems and Section 2.3 with the effects on the solvation structure of TiCl${}_{4}$. Section 2.4 contains experimentally performed Small-Angle X-Ray Scattering (SAXS) measurements with comparable system composition. The last section under the aspect of structural properties refers to the microheterogeneous structure of the investigated systems, their structural nature and linkage of polar and non-polar domains, as well as their percentage surface occupancy. For the dynamical properties, the mean square displacement of the different particles is first established in Section 2.5, in order to draw conclusions about the mobility and availability of the individual particles. In the following the lifetimes of different aggregates are listed and conclusions for the systems are also drawn. The final point of the results of Section 2.6 exhibits the calculated power spectra of the simulated systems and the power spectra of the individual ions. These are then compared with the experimentally determined Raman spectra of the same composition. Section 3 is followed by an all-encompassing discussion of all aspects of the investigated systems and the results are discussed. Section 4 Material and Methods explains the composition of the simulated systems and the setup of the AIMD simulations.

## 2. Results

In order to systematically investigate the underlying interactions we prepared four model systems. For a better comparison we also include the afore studied systems see Table 1, here denoted as I (IL mixture) and IW (IL plus water), which do not contain the TiCl${}_{4}$. Additionally, we define the systems IT (IL with TiCl${}_{4}$) and IWT (IL with TiCl${}_{4}$ and water). Table 1 shows the resulting simulation compositions. Please note, that we refer to the chloride anion ([Cl]${}^{-}$) also as Cl${}^{-}$, while with Cl we indicate the chlorine atom of the TiCl${}_{4}$ molecule.

### 2.1. Cation-Anion Interplay

**Figure 1:**
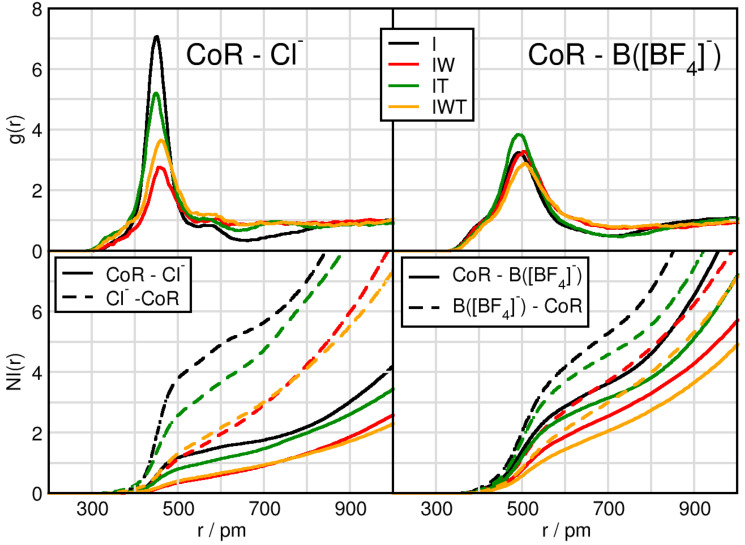
Radial distribution functions (RDFs) and number integrals (NIs) between the center of imidazolium ring CoR and chloride Cl${}^{-}$ (left panels), and the boron atom of tetrafluoroborate B([BF${}_{4}$]${}^{-}$) (right panels) in systems I (black), IW (red), IT (green) and IWT (orange).

We start by considering the interplay between the counterions through intermolecular radial distribution functions (RDFs) and number integrals (NIs), see Figure 1 and Table 2. While the RDFs give the probability of finding two sites apart normalized by the density, the number intergrals displays the amount of particles around each other. A more detailed derivation and description of the NI is presented in the analysis subsection. We consider the center of ring (CoR) and the central atom to the anions, Cl of [Cl]${}^{-}$ and B of [BF${}_{4}$]${}^{-}$. As a first observation the overall peak positions of the first maxima are shorter for the CoR-Cl${}^{-}$ distances than for the CoR-B(BF${}_{4}$) distances. A difference in the peak positions between the water-free and water containing systems is notable. Without water the CoR-anion distances are significantly shorter than in the moist systems, which is clearly indicated by the Δ I-IW and Δ IT-IWT values in Table 2. Here, the coordination number of anions around the cationic head groups (5th column in Table 2) decreases for both as seen in Δ I-IW and Δ IT-IWT by 1.6 for tetrafluoroborate and 1.8 and even 2.7 for chloride, respectively. This is a first evidence for water interrupting the typical cation-anion interplay.

Another obvious difference lies in the intensities of the first RDF maximum. Whereas this first RDF maximum of CoR-Cl${}^{-}$ function seem to be strongly dependent on the particular system, the CoR-B(BF${}_{4}$) behavior differs only little between the systems. Compared to system I the addition of TiCl${}_{4}$ (system IT) but especially the addition of water (systems IW and IWT) lead to a decreased CoR-Cl${}^{-}$ first RDF maximum. However, comparing I with IT indicates a slight shortening for both the CoR-Cl${}^{-}$ and CoR-B first maximum distance. This indicates that both TiCl${}_{4}$ and water competitively interact with chloride and thus disturb the cation-anion interplay. Through comparing CoR-Cl RDFs for systems IW (red) and IWT (orange) the slightly increased intensity of the orange curve additionally indicates that water and TiCl${}_{4}$ not only compete for the Cl${}^{-}$-cation interaction, but they also interact with each other, enabling the chloride to interact more intensely with the cation. Remarkable is also the decrease of the NI(r) for CoR-B when adding water and TiCl${}_{4}$ compared to system I.

Comparing the CoR-anions RDFs for each system separately, the strongest difference in cation-anion interplay occurs for system I. In this system the CoR-Cl first RDF maximum clearly overcomes the one of CoR-B(BF${}_{4}$), even though there are twice as many [BF${}_{4}$]${}^{-}$ anions in the system. This effect is also visible in system IT, but less pronounced, and even less pronounced in system IWT. System IW (red) is the only one in which the CoR-B(BF${}_{4}$) first peak is higher than the CoR-Cl${}^{-}$ one, again pointing towards strong water-chloride interactions. A more detailed observation of the water and TiCl${}_{4}$ effects will be considered in the following sections.

**Figure 2:**
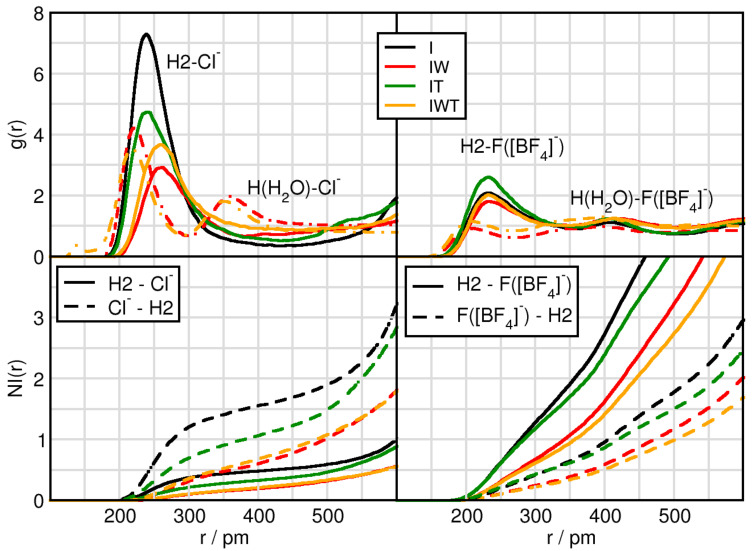
RDFs and NIs between H2 of the cation (solid) or H of H${}_{2}$O (dashed-dotted) and the contact atoms of the anion Cl${}^{-}$ (**left panels**) and the fluorine atoms of tetrafluoroborate F([BF${}_{4}$]${}^{-}$) (**right panels**) in systems I (black), IW (red), IT (green) and IWT (orange).

Since the hydrogen atoms directly bound to the imidazolium ring of the IL cations are acidic and, thus, undergo hydrogen-bonding, we now analyze the hydrogen bonding capabilities between the most acidic H2 hydrogen atom, which is located at the position of the carbon atom between the nitrogen atoms, and the two anions (in case of [BF${}_{4}$]${}^{-}$ now the fluor atoms are considered) present in the systems (c.f. Figure 2). Due to the previously observed strong influence of water the water-anion hydrogen bonding capabilities are taken into account as well, see Figure 2 dashed-dotted lines in the upper panels. Initial observations in system I exhibit that the peak positions of the first maxima of H2-F(BF${}_{4}$) are slightly shorter than the H2-Cl${}^{-}$ distances because fluorine has a smaller atom radius than chlorine. By adding water the hydrogen bonding between H2 and Cl${}^{-}$ is elongated from 239 pm to 259 pm, while the distance between H2-F(BF${}_{4}$) is unchanged. This shows that water interferes with the hydrogen bonding between chloride and the cation, in which chloride is solvated by water, whereas in the case of tetrafluoroborate it remains mostly unaffected, see also Table 2. Regarding the intensities of the first peaks the overall trend is very much comparable to the one we find for the anion-CoR interplay (c.f. Figure 1), indicating that the latter ones are strongly governed by the hydrogen bonding interaction between the counterions. Here again chloride illustrates a higher preference to form hydrogen bonds as compared to F(BF${}_{4}$) and especially water but also TiCl${}_{4}$ perturbs the hydrogen bonding in case of chloride. Taking into account the NIs a similar feature is being observed. While half a chloride anion surround the H2 atom of the cation, two F(BF${}_{4}$) accept a hydrogen bond. On the other hand, Cl${}^{-}$ sees 1.7 H2 atoms and each F(BF${}_{4}$) is sharing only 0.7 H2 atoms. Upon addition of water, the coordination number of H2 around F(BF${}_{4}$) is little reduced, the one of H2 around Cl${}^{-}$ decreases extremely, see Table 2. These reductions are also observed when adding TiCl${}_{4}$, but to a much lesser extend, i.e., considering the Δ I-IT and Δ IW-IWT values present that the effect of titanium tetrachloride on the H2-anion interaction is only small.

### 2.2. Solvation Structure of Water

**Figure 3:**
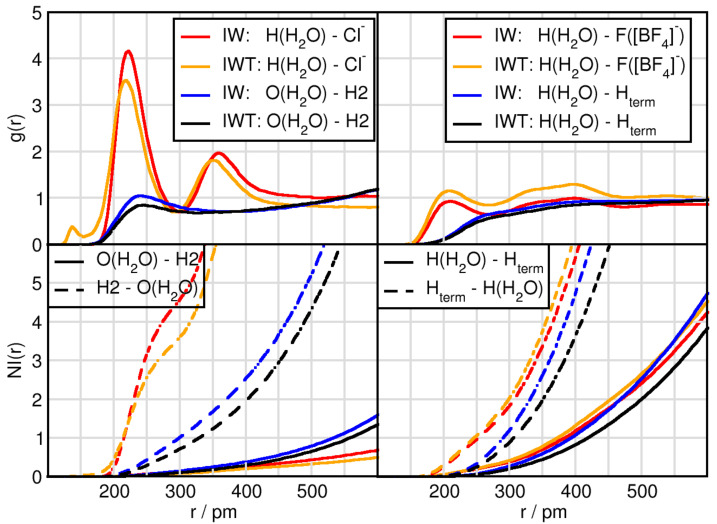
RDFs and NIs between H(H${}_{2}$O)/O(H${}_{2}$O) and the contact atoms of the anion Cl${}^{-}$ and H2 of the cation (**left panels**) and the fluorine atoms of tetrafluoroborate F([BF${}_{4}$]${}^{-}$) and terminal hydrogen atoms H${}_{\mathrm{term}}$ of the butyl side chain of the cation (**right panels**).

The results so far have focused on the effects of water and TiCl${}_{4}$ on the cation-anion interactions and their hydrogen bonding efficiency. In this subsection the solvation structure of water is analyzed. Comparing the peak position of the first maxima displays that the water-water distance is the shortest (c.f. Table 3 and Figure 3) followed by the H(H${}_{2}$O)-F([BF${}_{4}$]${}^{-}$ distance. While the fluorine atoms are surrounded by 1.3 protons of water which is slightly reduced upon TiCl${}_{4}$ addition, the Cl${}^{-}$ possess 4.6 protons as first solvent shell neighbors, which is reduced to 3.6 in the TiCl${}_{4}$ containing mixture. This underlines the fact that water qualitatively presents the strongest interaction with itself, followed by chloride, which is highly solvated. The high number of water molecules present in the system, lead to further influences with respect to the cation. While the H2-anion distance measured 259 pm and 232 pm for Cl${}^{-}$ and F([BF${}_{4}$]${}^{-}$ respectively, we observe a distance of 240 pm between the oxygen of water and the H2 of the cation.

### 2.3. Solvation Structure of TiCl${}_{4}$

**Figure 4:**
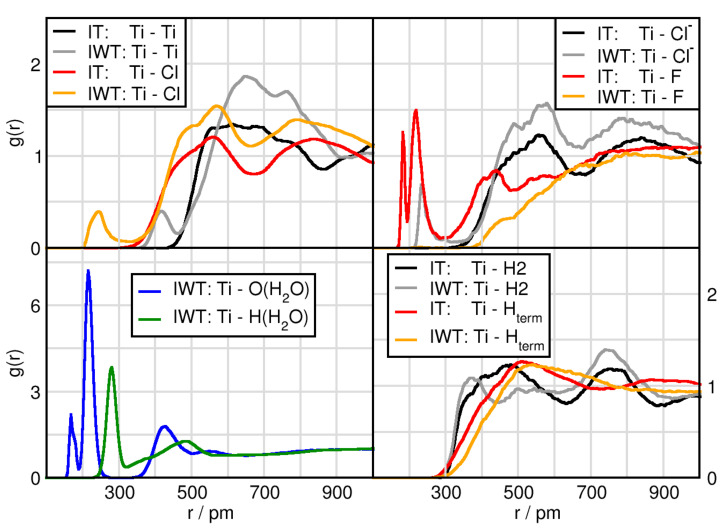
RDFs between the Ti(TiCl${}_{4}$) and atoms of the different compounds in systems IT and IWT. Please note the y-axis have different scales and that Cl marks the chlorine atom from TiCl${}_{4}$, while Cl${}^{-}$ stands for the chloride anion.

**Figure 5:**
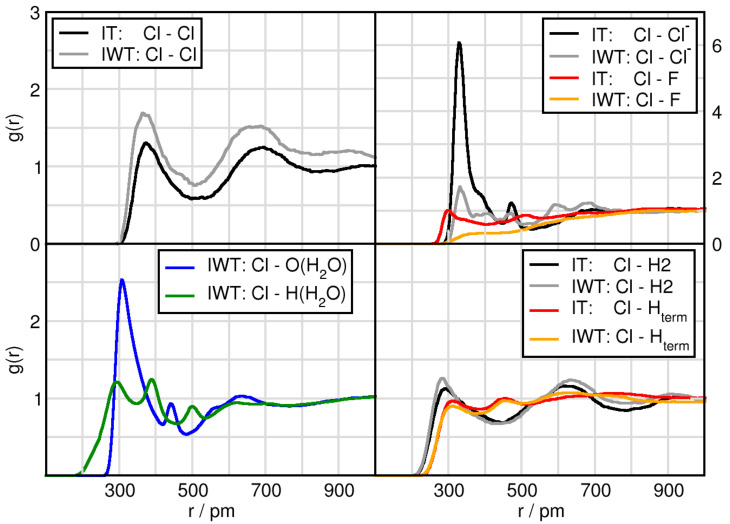
RDFs between the Cl(TiCl${}_{4}$) and all compounds in systems IT and IWT.

In this subsection we focus on the interactions related to the TiCl${}_{4}$, which are given in Figure 4 (Ti(TiCl${}_{4}$)) and Figure 5 (Cl(TiCl${}_{4}$)). In terms of solvation, TiCl${}_{4}$ seems to form solvent shells in the water free system at distances of Ti with Cl (550 pm), Cl${}^{-}$ (550 pm) and H2 (450 pm). These remain the same when adding water with the exception of H2 that obtains a shortened distance by approximately 50 pm and for Ti with O(H${}_{2}$O) (450 pm). Similarly, Cl of TiCl${}_{4}$ possesses a first solvent shell with other Cl atoms as well as with Cl${}^{-}$ anion at approximately 350 pm, with O(H${}_{2}$O) at 350 pm and with H2 at around 300 pm. There are several remarkable features in these RDFs. First of all, the orange and grey prepreak in the Ti-Ti (415 pm) and Ti-Cl (235 pm) functions indicate TiCl${}_{4}$ aggregation only occurring in the water containing system, Figure 4 (left upper panel). However, it should be noted that this is an isolated event in the course of the AIMD simulation. The right upper panel shows a grey (IWT: Ti-Cl${}^{-}$) prepeak and in red (IT: Ti-F) two peaks at short distances. These occurrences indicate the formation of titanium penta-complexes (formation of a trigonal bipyramid with three in-plane equatorial positions and two axial positions) with a behavior according to apicophilicity. While in the moist, the Cl${}^{-}$ anion can approach at the axial position (2nd distance), the fluorine atoms can approach both at the equatorial (1st peak) as well as at the axial position, but only if water is absent. Water can also occupy the equatorial and axial positions as seen in the lower left panel of Figure 4.

### 2.4. Small-Angle X-Ray Scattering (SAXS)

**Figure 6:**
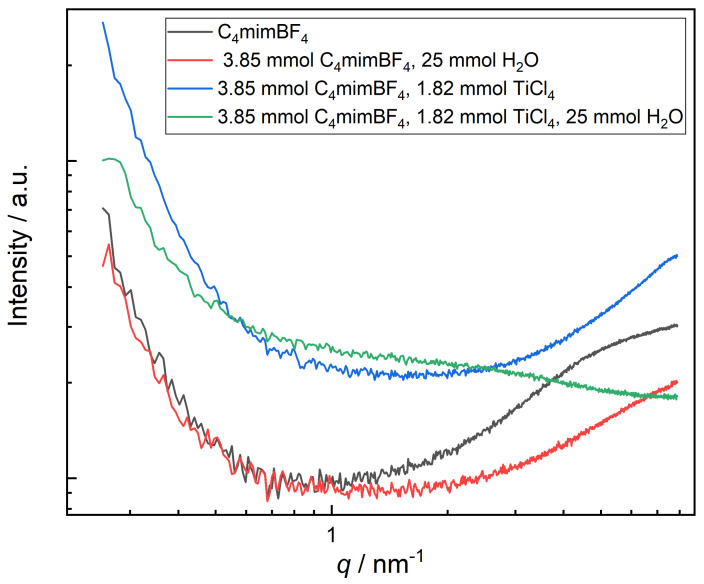
Experimental Small-Angle X-Ray Scattering (SAXS) measurement with comparable system compositions.

Small-Angle X-Ray Scattering (SAXS) was performed to study the possible formation of clusters or aggregates (Figure 6). The accessible q-range with a maximum value of q = 7.4 nm${}^{-1}$ allowed–in principle –the detection of objects down to about 1 nm in dimension. In general, the SAXS patterns shown in Figure 6 cannot be generated by particles, as there are no particles present in the measured solutions, and hence the different SAXS intensities must be interpreted in terms of dissolved molecular species (clusters). The four SAXS patterns show distinct differences: while samples I, IW and IT feature a rise in intensity at larger q (beyond 2 nm${}^{-1}$), sample IWT generates a different overall shape. Also, the samples IT and IWT both feature a much higher overall SAXS intensity, which is hence attributable to the scattering of Ti-containing species/clusters of ca. 1 nm in dimension. Furthermore, the SAXS pattern of sample IWT shows, in contrast to IT, a broad scattering feature at q ca. 1–3 nm${}^{-1}$ which can be interpreted as the form factor of aggregates of titanium-complexes of ca. 1–2 nm, which are formed upon the interaction with water. A quantitative evaluation of these SAXS data is not possible, but they already indicate the presence of objects in the nanometer range. In this respect, SAXS supports the special behavior of the IWT system as indicated by the theoretical calculations.

### 2.5. Domain and Voronoi Analysis

Using the domain and voronoi analysis, we divide the molecules and molecular functional groups of our investigated systems into subsets. These subsets are analyzed with respect to their connectivity and their neighborhood behavior. If the domain exhibits a value of one, the microphase is completely interconnected and by larger numbers these subsets are more dispersed.

Table 4 displays the media divided into polar (P: all polar parts), nonpolar (NP: parts of the butyl side chain), the TiCl${}_{4}$ molecules as its own subset and if present water. Previous studies have shown, [5,6,8,23] that in some neat [C${}_{n}$C${}_{m}$Im] based ILs, both polar and nonpolar domains are completely connected. This structural orientation is also observed in system I, while all other systems do not, with the exception of the polar domain of system IT. Keeping in mind the immense amount of H${}_{2}$O compared to TiCl${}_{4}$, the perturbation of the microheterogeneous systems by water but also by TiCl${}_{4}$ is obvious. Water perturbs both the polar and also the nonpolar parts, while TiCl${}_{4}$ addition mainly alters the nonpolar microphase.

In this context Table 5 presents a further subdivision of the polar domain in three separate subsets of the imidazolium ring, [BF${}_{4}$]${}^{-}$ and [Cl]${}^{-}$. In the case of system IT, the cation ring subset has an average of 1.1, while those of [BF${}_{4}$]${}^{-}$ and [Cl]${}^{-}$ are 9.1 and 8.9, indicating an almost fluid connection between the polar parts of the cation and a strong dispersion of the polar anions in system IT. In system IWT, the subdivision of the polar domain has even more influence on the dispersion of the polar subsets than in system IT.

Further insight in this respect is provided by the analysis of the surface coverage from Table 6. Obviously, there is a high surface coverage by the butyl side chain due to chemical bonding to the cation ring, which is the most common ionic species. In system I, the [BF${}_{4}$]${}^{-}$ together with other ring subsets mainly cover the surface of the cationic ring, while the ring majorly covers the surface of both the anions with the larger extend for the Cl${}^{-}$ anions. The [BF${}_{4}$]${}^{-}$ shares its surface with the nonpolar side chain, illustrating its hydrophobicity.

The most severe change is the cationic ring coverage reduction observed for the anions and the subsequent coverage by water. Also the cationic ring and the side chain loose anionic coverage. These effects are also observed although less severe in system IT where TiCl${}_{4}$ is added. Interestingly this extreme reduction when water is added for the anion is further increased by adding also TiCl${}_{4}$ (system IWT) with the exception of the Cl${}^{-}$ anion. In the system with all components (IWT) the surface coverage of the Cl${}^{-}$ anions by the cationic rings changes back to a higher one from 25.5% compared to the moist system (IW) to 30.6%. In all systems, where water is present it is mainly covered by water itself. TiCl${}_{4}$ is mainly covered by the cationic rings together with the side chains, but also by other TiCl${}_{4}$ molecules. Nevertheless, the anions achieve a sizeable surface coverage especially when compared to the coverage by themselves. The situation changes again when all components are present (IWT), all coverages are reduced and replaced by water.

### 2.6. Dynamical Properties

In the next part we investigate dynamical properties to estimate the mobility and availability of the particles. By “availability” we refer to availability of the particles as possible reactants.

**Figure 7:**
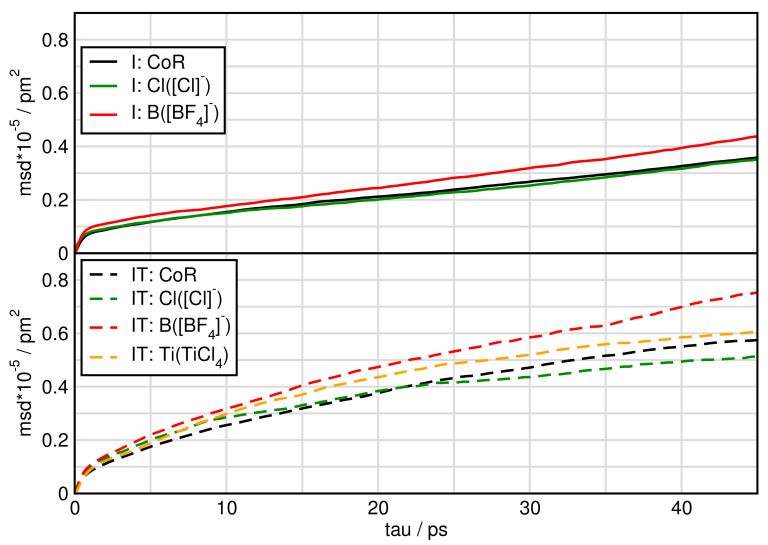
Mean square displacement of particles in systems I and IT.

**Figure 8:**
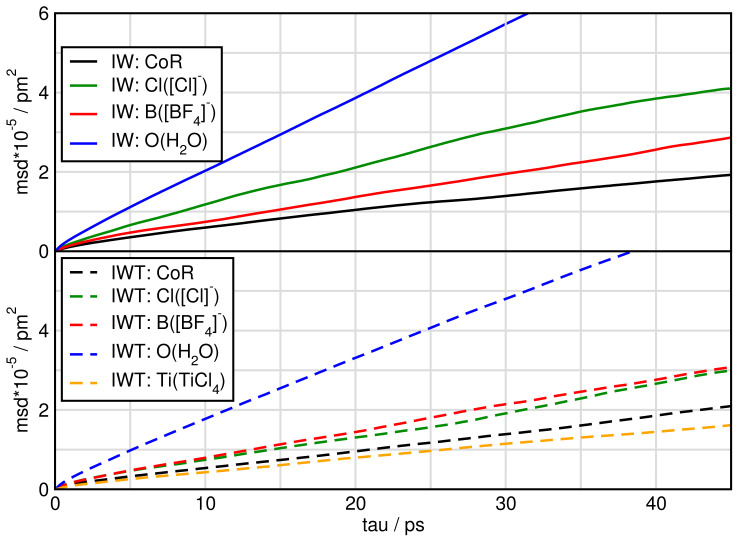
Mean square displacement of particles in systems IW and IWT.

In Figure 7 and Figure 8 we plot the mean square displacement of all particles in the different systems. For system I, it is visible that all ions diffuse approximately the same way with a little faster movement of [BF${}_{4}$]. In the pure ionic liquid [C${}_{4}$C${}_{1}$Im][BF${}_{4}$] Watanabe and coworkers [63] found the same diffusivity of the cation compared to the anion. Comparing system I and IT we find, that TiCl${}_{4}$ increases the mobility of the particles.

When water is added the mean square displacement raises by approximately an order of magnitude. Focusing on Figure 8 of the systems including water shows that most species of system IW are faster than of system IWT. In both IW and IWT water is the fastest species, but now the [Cl]${}^{-}$ anions are next followed by the [BF${}_{4}$]${}^{-}$ anions, while the cations and the TiCl${}_{4}$ molecules are relatively slow. This indicates that while the [Cl]${}^{-}$ anions are dominated by the cations movements in system I, as soon as water molecules are present, they influence the mobility of the [Cl]${}^{-}$ anions.

Also the lifetimes of certain species, can be investigated for AIMD simulations. For this purpose, continuous autocorrelation functions were produced for specific aggregates.

**Figure 9:**
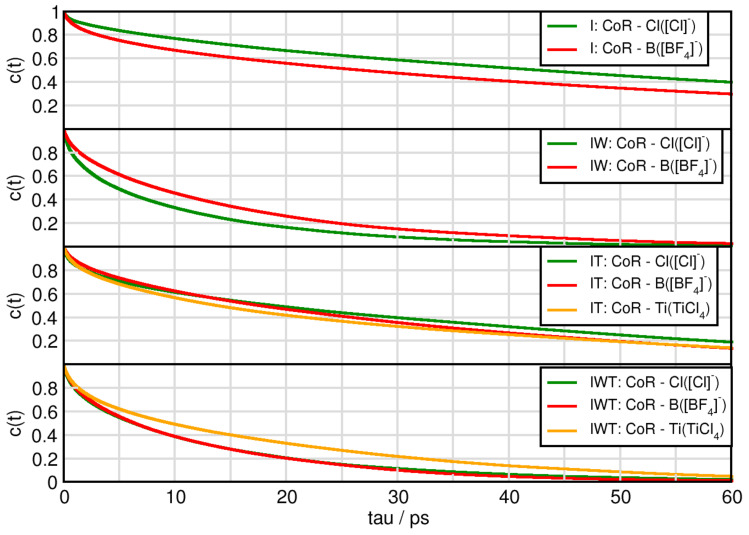
Continuous autocorrelation of aggregates associated with the cation. Aggregates were determined at approximately their first minimum in the RDF.

The lifetimes of ion pairs between the cation and chloride behave according to the following trends (c.f. Figure 9): System I has the longest duration, followed by IT, then IWT and finally IW. In case for chloride aggregates it is not surprising that through addition of water these aggregates have shorter lifetimes, as there is a strong interference of water with chloride. The imidazolium-tetrafluoroborate aggregates almost show the same trends. Interestingly, while the aggregates between the cation and the [Cl]${}^{-}$ anions are longer lived in the pure ionic liquid mixture, adding water reverses this behavior while adding TiCl${}_{4}$ molecules makes both cation-anion behaviors similar. Aggregates between the cation and TiCl${}_{4}$ in system IT possess longer lifetimes than in system IWT. Overall all cation aggregates are in the same order of magnitude.

**Figure 10:**
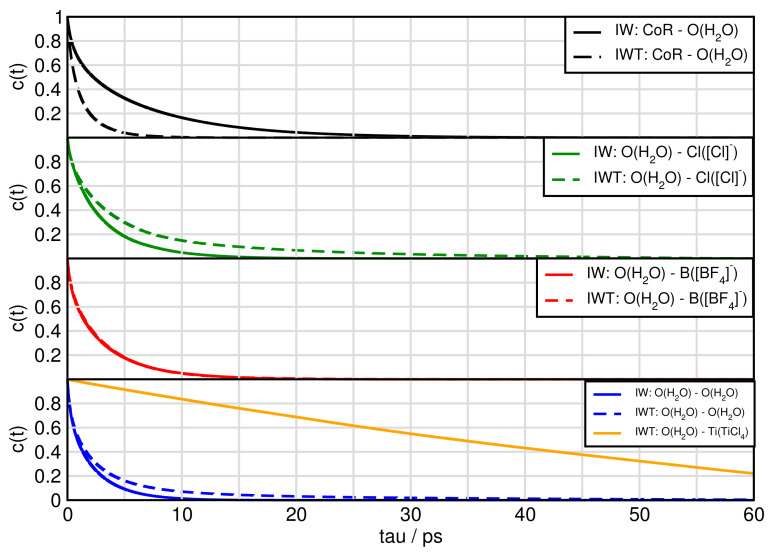
Continuous autocorrelation of aggregates associated with water. Aggregates were determined at approximately their first minimum in the RDF.

**Figure 11:**
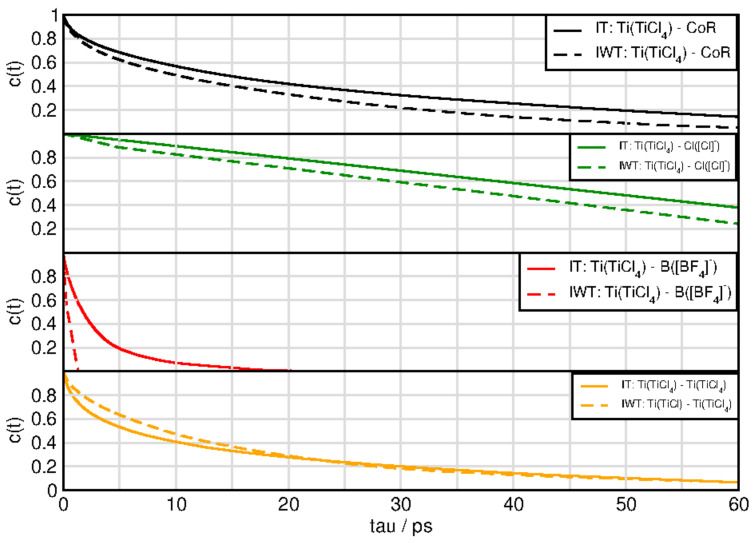
Continuous autocorrelation of aggregates associated with titaniumtetrachloride. Aggregates were determined at approximately their first minimum in the RDF.

Figure 10 and Figure 11 exhibit the aggregates with water and titanium tetrachloride. Compared to the ion-ion and ion-TiCl${}_{4}$ in the water free-systems all aggregates break much faster with the strong exception of the water-TiCl${}_{4}$, indicating a correlation to the strong interaction between these two species. Similarly high is the life time of the TiCl${}_{4}$-Cl${}^{-}$ complex and the self-aggregation of TiCl${}_{4}$ also exihibts a sizeable life time. The TiCl${}_{4}$-[BF${}_{4}$]${}^{-}$ is very short lived in IWT and only in the water-free situation increases a bit, which explains the low probability to form reactions with this species at least in the moist system. The formation of the fluoro-titanium compounds might be preferred under water-free conditions.

For system IWT the following trends are observed. Water-TiCl${}_{4}$ species demonstrates the longest lifetimes followed by water-chloride and finally water-imidazolium and water-water aggregates. For the aggregates formed with TiCl${}_{4}$ and the following trends are observed: The titanium tetrachloride—chloride species appear with the longest lifetimes followed by the TiCl${}_{4}$-water species. Afterwards the cation aggregates follow, then the self-aggregated TiCl${}_{4}$ species and finally the BF${}_{4}$ species.

All this observations from the mean square displacement (msd) and autocorrelation functions of specific aggregates into account show, that on the one hand mobile and available particles, such as water, chloride and [BF${}_{4}$]${}^{-}$.

### 2.7. Spectroscopy

In order to obtain vibrational spectra from AIMD, power spectra based on nuclear velocities are discussed. The obtained power spectrum contains peaks for each normal mode vector and gives insights into vibrational frequencies independent of selection rules, such as in IR and Raman spectra. These are then compared with experimentally obtained Raman spectra with the same system composition.

**Figure 12:**
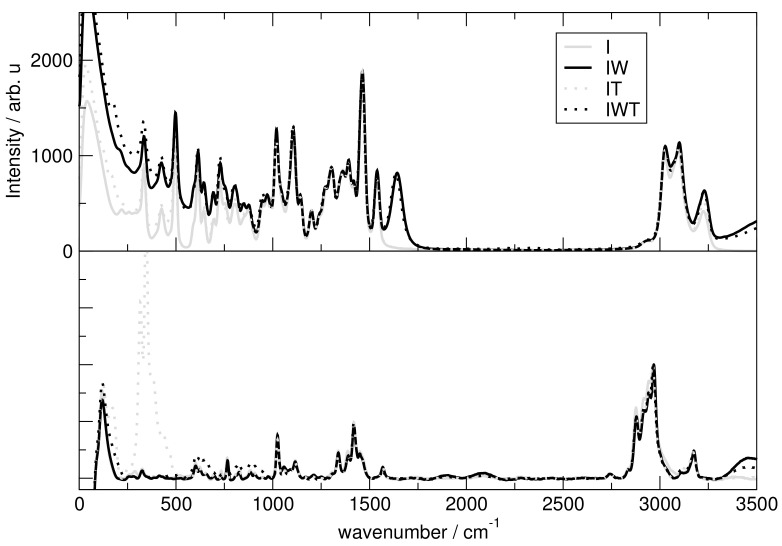
Upper panel: Complete power spectrum calculated for system I (solid line, gray), IW (solid line, black), IT (dotted line, gray) and IWT (dotted line, black) Lower panel: Experimental Raman spectrum with the same mixing ratio as theoretical systems.

**Figure 13:**
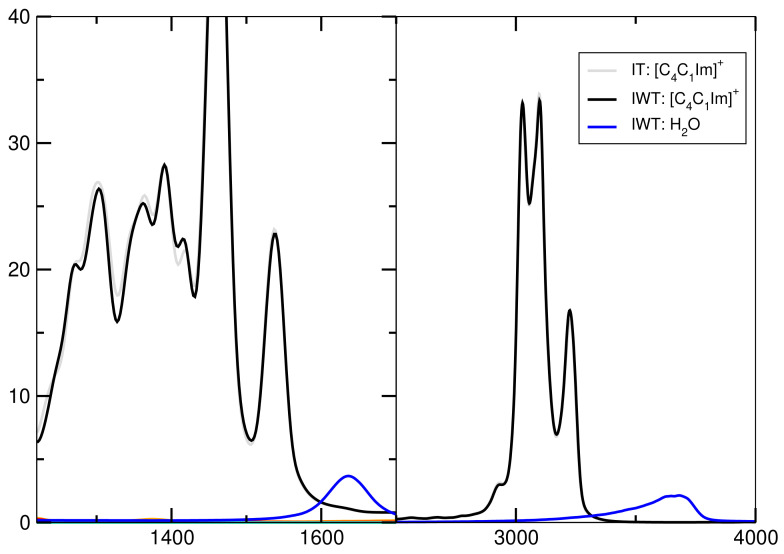
Power spectrum for high wavenumbers.

**Figure 14:**
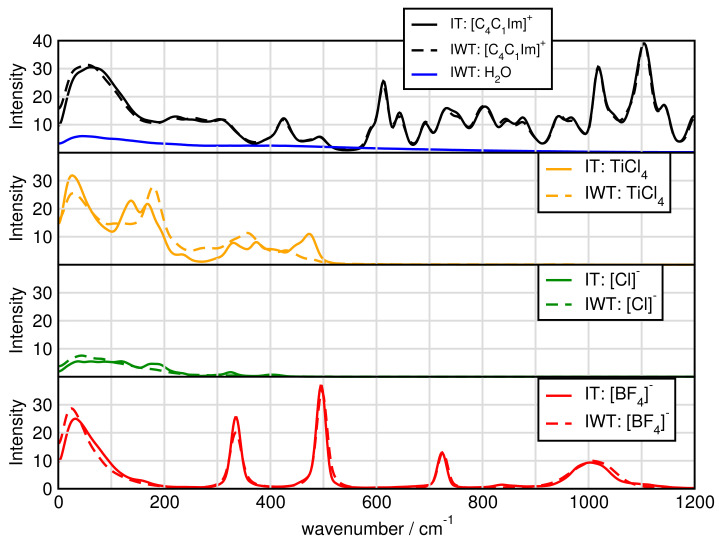
Power spectrum for particular ions.

Figure 12 presents the spectra for all investigated systems generally having similar shapes. Systems including H${}_{2}$O have three significant characteristics. First, it exhibits high intensities in the low-wavenumber region related to the heavy atom modes, also depict in Figure 12 and Figure 13 for the particular particles. Second, the water binding modes at around 1640 cm${}^{-1}$ and third the stretching modes of O-H at 3300 cm${}^{-1}$. Also signals for tetrafluoroborate up to 1200 cm${}^{-1}$ are observed. Figure 14 shows that the addition of water causes the signals below 500 cm${}^{-1}$ to lose intensity. Also cation breathing modes in the low wavenumber are observed. In the range of 500–1000 cm${}^{-1}$ vibrational modes of carbon atoms are seen. Signals at 2800 and 3000 cm${}^{-1}$ relate to the butyl sidechain reflecting the stretching and Fermi resonance modes of methylen—and methyl-groups [64,65]. Also vibration modes of C-H of the cationic ring are observed at 3000 and 3200 cm${}^{-1}$ [64,65]. The comparison between the calculated and experimental spectra shows a satisfactory global agreement of the shape of the spectra and a redshift at high wave numbers of the theoretical spectra.

## 3. Discussion

In this study we investigated the complex molecular-level solvation effects within multi-component ionic liquid (IL) systems ([C${}_{2}$C${}_{1}$Im][Cl][BF${}_{4}$]) mixed with TiCl${}_{4}$ and water by performing state of the art ab initio molecular dynamics (AIMD) simulations. We distinguish four systems: I) IL mixture, IW) IL mixture plus water, IT) IL mixture with TiCl${}_{4}$, ITW) IL mixture with TiCl${}_{4}$ and water.

First investigations focused on the structural properties of the investigated systems. Therefore, radial distribution functions (RDFs) and number integrals (NIs) were evaluated. By taking into account the peak positions of the first maxima, their intensities and relative ratios indicate that both TiCl${}_{4}$ and water disturb the typical cations-anion interactions. Upon addition of water or TiCl${}_{4}$, the cation-anion interactions for both [Cl]${}^{-}$ and [BF${}_{4}$]${}^{-}$ are weakened, which can be concluded from a notable shift of the first peak maxima in the cation-anion RDFs towards higher distances. This effect is stronger in case of [Cl]${}^{-}$, which is also visible from first peak intensities. These decrease drastically for [Cl]${}^{-}$ when adding water or TiCl${}_{4}$, whereas those for [BF${}_{4}$]${}^{-}$ differ only little between the different systems. However, comparing system IW and IT to IWT reveals that TiCl${}_{4}$ and water do not only compete for the chloride anion, but also interact with each other, enabling a stronger interaction between [Cl]${}^{-}$ and the cation.Closer observations show that the cation-anion interactions are mostly dominated by h-bonding between the acidic hydrogen atoms directly bound to the imidazolium ring of the cation.

Focusing on the solvation structure of water it was found that the water-water self-interactions are dominant, followed by chloride, which is strongly solvated, while the water-BF${}_{4}$ and water-cation interactions are rather weak.

Investigating the solvation structure for TiCl${}_{4}$, remarkable features can be observed. In case of system IWT, prepeaks in the Ti-Ti and Ti-Cl (where Cl marks the chlorine atom from other TiCl${}_{4}$ molecules) RDFs might indicate TiCl${}_{4}$ aggregation, which is not visible in the water-free system IT. Moreover, prepeaks in the RDFs between Ti-[Cl]${}^{-}$, and Ti-O(H${}_{2}$O) in system IWT and between Ti-F(BF${}_{4}$) in system IT, point towards the formation of Ti-penta-complexes, which follow the phenomenon of apicophilicity. In the case of chloride, we observe a coordination to the axial position of Ti, while the fluoride and water ligands can coordinate both axially and equatorially.

Performing a Voronoi analyses and investigating the surface coverage of the different relevant system units, exhibits that water has a greater influence on the microheterogeneous structure of the systems than TiCl${}_{4}$. However, the high number of water molecules should be taken into account. Overall, the polar network of the ionic liquid collapses when TiCl${}_{4}$ and water are added. Chloride leaves the polar domain of the ionic liquid mixture and enters the water domain, while [BF${}_{4}$]${}^{-}$ remains unchanged. This observation seriously affects the interaction of the ionic liquid network.

Investigations on the dynamical properties of the systems comprised the mean square displacement (msd) of the particles and the autocorrelation functions of specific aggregates. Analyzing the msd-curves show that upon addition of both water and TiCl${}_{4}$, the motion of all particles increases. Especially the motion of [Cl]${}^{-}$ is strongly increased by the addition of water. This is also reflected in the short lifetime of the aggregates in which the chlorides are involved. In general it can be stated that the aggregates in the system I have the longest lifetime, followed by system IT, IWT and finally IW. The aggregates formed with water and TiCl${}_{4}$ yield shorter lifetimes compared to the ion-ion and ion-TiCl${}_{4}$ lifetimes, but the specific aggregates between H${}_{2}$O and TiCl${}_{4}$ show a high longevity. This indicates a high reactivity between these species. Likewise, long lifetimes are observed for aggregates of TiCl${}_{4}$-[Cl]${}^{-}$ and TiCl${}_{4}$ with itself. Noteworthy is the fact that the aggregate between TiCl${}_{4}$ and [BF${}_{4}$]${}^{-}$ in system IWT has a quite short lifetime, showing that the probability is rather low that this species would react in the moist system and is favored in water free system. All these observations of the structural properties, imply, that pre-steps of chemical reactions take place. In the last section of the results the calculated power spectra for the whole systems as well as for the single components, respectively, are provided, which can be compared to experimental vibrational spectra of these compositions.

## 4. Materials and Methods

### 4.1. Composition and Preparation of Simulation Boxes

To generate the simulation boxes for the pre-equilibration of the systems via classical MD simulations first the single molecules were built using the software package MOLDEN (version 5.4) [66]. In order to derive the initial starting geometries of the simulation boxes, the program PACKMOL (version 16.228) was used [67,68].

### 4.2. Pre-equilibration via Classical MD Simulations

In order to pre-equilibrate each system, classical MD simulations are carried out using the molecular dynamics simulator LAMMPS (version from the 14 May 2016) [69]. Periodic boundary conditions are applied to resemble bulk conditions. For the description of the IL cations and anions intra- and intermolecular interactions the force field parameters were chosen according to Canongia Lopes and Pádua, who developed the parameters based on the OPLS-AA/AMBER framework and on the OPLS-AA model, which is oriented toward the calculation of equilibrium thermodynamic and structural properties [70,71]. The charges of the cations and anions were scaled by a factor of 0.8 since this has shown a more reasonable representation of mass transport properties of ILs. In order to obtain Lennard-Jones cross terms, the Lorentz-Berthelot mixing rules were applied [72,73]. All non-bonded interactions between the atoms were restricted to a cutoff radius of 16 Å. The simulations were performed at a temperature of 380 K and a pressure of 1 bar (see experimental setup), which was controlled by applying the Nosé–Hoover chain thermostat and the Nosé–Hoover barostat, respectively, using a time step of 0.5 fs [74,75,76].

The pre-equilibration runs were composed of several steps, which are described briefly in the following: First, a run over 300,000 steps in the NVE ensemble was performed in which the starting volume from packmol was adjusted to the corresponding experimental density provided by the experimentalists (1.2942 g cm${}^{-3}$ for system IT and 1.1943 g cm${}^{-3}$ for system IWT). Subsequently, another 300,000 steps were carried out in the NVE ensemble to equilibrate the system on the experimental densities. Next, the systems were equilibrated over 1,000,000 steps in the NVT ensemble and finally, two trajectories are produced: The first one over 1,000,000 steps printing out the atoms coordinates every 1000th step (long trajectory), and the second one over further 100 steps printing out the atoms coordinates every 10th step (short trajectory).

**Figure 15:**
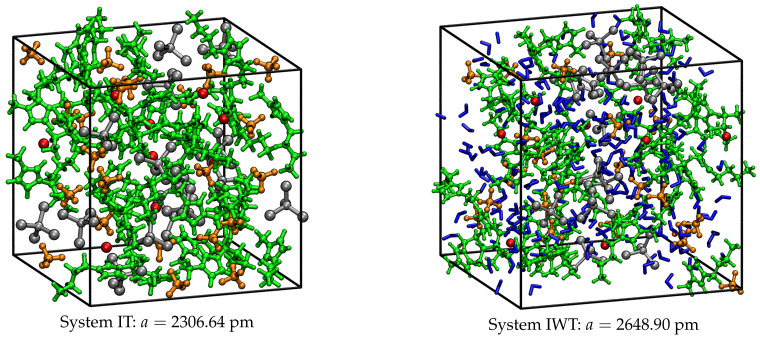
Simulation boxes for systems IT and IWT and corresponding box length *a* in pm after pre-equilibration. Within the boxes the IL cations are colored in green, [BF${}_{4}$]${}^{-}$ anions in orange, [Cl]${}^{-}$ anions in red, [TiCl${}_{4}$] in silver and the water molecules in blue.

After these steps, the systems can be considered as fully equilibrated. For the subsequent AIMD simulations, the atom coordinates of the last time step from the short trajectory were considered as starting geometries. Figure 15 presents the resulting boxes with their corresponding box length *a* in pm.

### 4.3. AIMD Simulations

The AIMD simulations were carried out using density functional theory (DFT) with the CP2K [77] program package applying the QUICKSTEP module [78]. Here, the hybrid Gaussian and plane waves (GPW) approach is used to calculate the energies and forces on the atoms. The molecularly optimized short range double-ζ basis set (MOLOPT-DZVP-SR-GTH) [79] was applied to all atoms together with the revPBE hybrid functional and the corresponding PBE Goedecker–Teter–Hutter pseudopotentials for core electrons [80,81,82]. A 400 Ry density CUTOFF criterion with the finest grid level was employed, together with multigrids number 5 (NGRID 5 and REL_CUTOFF 30) using the smoothing for the electron density (NN10_SMOOTH) and its derivative (NN10) [78]. Dispersion interactions were accounted for by using the DFT-D3 type of a pair potential van der Waals density functional [83,84]. For the SCF calculation, a value of 1.0 $\times \;10^{-6}$ was used as target accuracy for the SCF convergence (EPS_SCF 1.0 $\times \;10^{-6}$). The DIIS minimizer [77] was used to reach a faster orbital transformation via direct inversion in the iterative subspace. The maximum number of SCF iterations to be performed for one iteration was set to 100, while for the outer SCF loops a maximum number of 10 was taken into account. Periodic boundary conditions were applied to avoid boundary/edge effects and thus simulate a bulk-like situation. The simulations were performed under the canonical NVT ensemble, at a temperature of 380 K (see experimental setup), which was controlled by applying the Nosé–Hoover chain thermostat with a time constant of 50 fs [74,75,76]. The simulation time step amounted for 0.5 fs.

In a 5 ps equilibration run the systems was given time to relaxate using the REGION MASSIVE keyword, which applies a thermostat for every single atom in order to achieve a faster equilibration. For the equilibration, the density CUTOFF criterion was decreased to 200 Ry and a value of 1.0 $\times \;10^{-5}$ was used for the SCF convergence (EPS_SCF 1.0 $\times \;10^{-5}$).

The subsequent production runs comprised 205,212 time steps (≈103 ps) for system IT and 180,133 time steps (≈90 ps) for system IWT.

### 4.4. Small-Angle X-Ray Scattering (SAXS)

SAXS experiments were carried out at Elettra Synchrotron Radiation facility (Elettra-Sincrotrone Trieste; AREA Science Park; 34149 Basovizza, Trieste, Italy) using the SAXS Beamline 5.2. For the measurements a laser with a wavelength of 0.077 nm was used and a Pilatus3 1M detector (Dectris, Baden-Daetwill) was used for detection. The samples were filled into glass capillaries (80 mm × 1.5 mm; Hilgenberg GmbH, Malsfeld, Germany) and heated to 95 ${}^{\circ }$C.

### 4.5. Raman Spectroscopy

Raman spectroscopy measurements were carried out using a SENTERRA Raman spectometer (Bruker Optics). During the measurements a MPlan 20× objective was used in combination with a Bruker A697-S 90${}^{c}irc$ adapter. The laser power at 532 nm was 5 mW and the samples were heated to 95 ${}^{\circ }$C.

### 4.6. Analysis

Our open source program TRAVIS (Trajectory Analyzer and Visualizer) is used to examine the simulated systems [85]. This powerful tool enables the time-dependent investigation of structural features of the solvent and the solute, intra- and intermolecular interactions within or between the components and the elucidation of chemical reaction mechanisms by calculating different distribution functions (DFs) between relevant atoms and positions of the observed systems. The most often used features are radial distribution functions (RDFs) and number integrals (NIs). The NI is obtained from the integral of g(r) and is as follows:

(1) $$N_{c}=4\pi \rho \int_{0}^{r_{min}}r^{2}g\left(r\right)dr$$

N${}_{c}$ is the number of particles within the first solvation shell. These are derived from the integral limits from 0 to r${}_{min}$ the first minimum of g(r) and ρ the density of the system under investigation. The integral limits up to the first minimum reflect the coordination numbers of the first neighbors. In order to quantitatively evaluate the structuring in the systems we additionally perform the domain analysis on the basis of the Voronoi tessellation. The mean square displacement (msd) of all components in the systems are calculated to evaluate the transport properties of the compounds. Vibrational spectra are also generated with TRAVIS.

## Figures and Tables

**Table 1:** Ball-and-stick representations and number of molecules composing the four simulated systems I, IW, IT and IWT.

System	[C${}_{4}$C${}_{1}$Im]${}^{+}$	[BF${}_{4}$]${}^{-}$	[Cl]${}^{-}$	TiCl${}_{4}$	H${}_{2}$O
	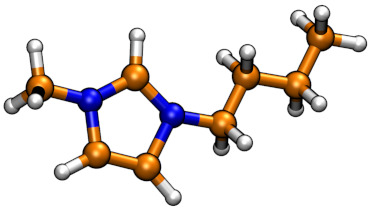	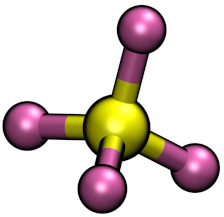	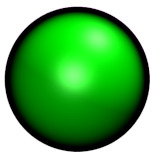	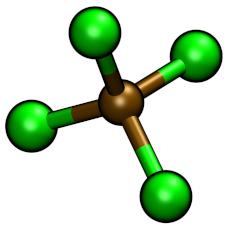	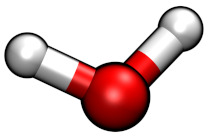
I	32	22	10	−	−
IW	32	22	10	−	211
IT	32	22	10	15	−
IWT	32	22	10	15	211

**Table 2:** Distance of first RDF maximum and minimum as well as value of NIs at the first minimum, $\bar{\mathrm{NI}}$ gives the value for the atom combination vice versa.

	r(CoR-Cl${}^{-}$)	NI	$\bar{\mathrm{NI}}$	r(H2-Cl${}^{-}$)	NI	$\bar{\mathrm{NI}}$
	r/pm			r/pm		
	**max min**			**max min**		
I	452 700	1.8	5.6	239 455	0.5	1.7
IW	458 700	0.9	2.9	259 402	0.1	0.4
IT	449 700	1.5	4.8	240 455	0.4	1.3
IWT	462 700	0.9	3.0	257 402	0.2	0.7
Δ(I-IW)	−6 0	0.9	2.7	−20 53	0.4	1.3
Δ(IT-IWT)	−13 0	0.6	1.8	−17 53	0.2	0.6
Δ(I-IT)	3 0	0.3	0.8	−1 0	0.1	0.4
Δ(IW-IWT)	−4 0	0.0	−0.1	2 0	−0.1	−0.3
	**r(CoR−B([BF${}_{4}$]${}^{-}$))**	**NI**	$\bar{\mathrm{NI}}$	**r(H2-** **F([BF${}_{4}$]${}^{-}$))**	**NI**	$\bar{\mathrm{NI}}$
	**r/pm**			**r/pm**		
	**max min**			**max min**		
I	492 700	3.6	5.3	233 355	2.0	0.7
IW	502 700	2.5	3.7	232 330	0.9	0.3
IT	490 700	3.1	4.6	233 355	1.8	0.6
IWT	505 700	2.0	3.0	232 330	0.8	0.3
Δ(I-IW)	−10 0	1.1	1.6	1 25	1.1	0.4
Δ(IT-IWT)	−15 0	1.1	1.6	1 25	1.0	0.3
Δ(I-IT)	2 0	0.5	0.7	0 0	0.2	0.1
Δ(IW-IWT)	−3 0	0.5	0.7	0 0	0.1	0.0

**Table 3:** Distance in pm of first RDF maximum and minimum as well as value of NIs at the first minimum, $\bar{\mathrm{NI}}$ gives the value for the atom combination vice versa.

	r(O(H${}_{2}$O)-H2)				O(H${}_{2}$O)-H(H${}_{2}$O)				r(O(H${}_{2}$O)-O(H${}_{2}$O)			
	**max**	**min**	**NI**	$\bar{\mathrm{NI}}$	**max**	**min**	**NI**	$\bar{\mathrm{NI}}$	**max**	**min**	**NI**	$\bar{\mathrm{NI}}$
IW	240	400	0.4	2.6	186	254	−	−	286	400	4.6	4.6
IWT	245	400	0.3	2.0	184	254	−	−	284	400	4.0	4.0
Δ(IW-IWT)	5	0	−0.1	−0.6	2	0	−	−	2.0	0.0	0.6	0.6
	**r(H(H** ${}_{2}$ **O)-H** ${}_{\mathrm{term}}$ **)**				**r(H(H** ${}_{2}$ **O)-Cl** ${}^{-}$ **)**				**r(H(H** ${}_{2}$ **O)-F([BF** ${}_{4}$ **]** ${}^{-}$ **)**			
	**max**	**min**	**NI**	$\bar{\mathrm{NI}}$	**max**	**min**	**NI**	$\bar{\mathrm{NI}}$	**max**	**min**	**NI**	$\bar{\mathrm{NI}}$
IW	−	−	−	−	222	300	0.1	4.6	210	270	0.3	1.3
IWT	−	−	−	−	219	300	0.1	3.6	206	270	0.3	1.4
Δ(IW-IWT)	−	−	−	−	−3	0	0	1.0	4	0	0	−0.1

**Table 4:** Domain Voronoi analysis for systems I, IW, IT and IWT. Dissection into polar (P: ring, methyl group, anions), nonpolar (NP:butyl chain), TiCl${}_{4}$ and water (H${}_{2}$O).

	P	NP	TiCl${}_{4}$	H${}_{2}$O
I	1.0	1.0	−	−
IW	1.2	5.3	−	1.2
IT	1.0	1.5	1.4	−
IWT	1.1	5.4	2.3	1.1

**Table 5:** Domain Voronoi analysis for systems IT and IWT. Dissection into ring (ring, methyl), chain (butyl chain), [BF${}_{4}^{-}$], [Cl${}^{-}$], TiCl${}_{4}$ and water (H${}_{2}$O).

	ring	chain	[BF${}_{4}$]${}^{-}$	[Cl]${}^{-}$	TiCl${}_{4}$	H${}_{2}$O
IT	1.1	1.5	9.1	8.9	1.4	−
IWT	2.0	5.4	13.8	9.3	2.3	1.1

**Table 6:** Surface coverage from Voronoi analysis for system I, IW, IT and IWT. The subsets are ring (ring of the cation plus methyl group plus the CH${}_{2}$ space to the longer side chain), C${}_{3}$H${}_{7}$ (the propyl rest of butyl side chain), [BF${}_{4}$]${}^{-}$, [Cl]${}^{-}$,TiCl${}_{4}$ and water. ${}^{★}$ indicates referred coverage, columns = observed surface (values given are in percentages).

	Ring ${}^{★}$	C${}_{3}$H${}_{7}^{★}$	[BF${}_{4}$]${}^{-★}$	[Cl]${}^{-★}$	TiCl${}_{4}^{★}$	H${}_{2}$O${}^{★}$
	system I					
ring	27.8	49.8	65.3	81.0	−	−
C${}_{3}$H${}_{7}$	35.7	28.9	31.3	16.8	−	−
[BF${}_{4}$]${}^{-}$	28.4	19.0	3.0	1.8	−	−
[Cl]${}^{-}$	8.1	2.3	0.4	0.4	−	−
	system IW					
ring	12.3	36.5	39.0	25.5	−	20.0
C${}_{3}$H${}_{7}$	25.9	15.9	17.4	10.7	−	12.0
[BF${}_{4}$]${}^{-}$	16.8	10.5	2.2	0.6	−	8.4
[Cl]${}^{-}$	2.5	1.5	0.1	0.0	−	2.9
H${}_{2}$O	42.5	35.6	41.3	63.2	−	56.7
	system IT					
ring	19.7	41.0	58.1	61.9	41.3	−
C${}_{3}$H${}_{7}$	29.5	24.0	25.8	13.6	26.8	−
[BF${}_{4}$]${}^{-}$	25.1	15.5	3.0	2.4	11.4	−
[Cl]${}^{-}$	6.0	1.8	1.0	0.8	4.4	−
TiCl${}_{4}$	19.7	17.7	12.6	21.3	16.2	−
	system IWT					
ring	9.1	34.5	32.2	30.6	27.1	17.1
C${}_{3}$H${}_{7}$	24.6	13.6	15.9	10.6	17.0	9.9
[BF${}_{4}$]${}^{-}$	13.8	9.5	1.5	1.3	2.3	9.5
[Cl]${}^{-}$	3.0	1.5	0.3	0.3	1.0	2.4
TiCl${}_{4}$	12.7	11.2	2.5	4.9	12.2	8.8
H${}_{2}$O	36.8	29.7	47.6	52.3	40.5	52.3

## Data Availability

The data presented in this study are available in this article.

## Abbreviations

The following abbreviations are used in this manuscript:

IL	ionic liquid
AIMD	ab initio molecular dynamics
RDF	radial distribution function
NI	number integral
CoR	center of ring
IR	infrared
SAXS	small angle X-ray scattering
